# Astaxanthin and eicosapentaenoic acid production by *S4*, a new mutant strain of *Nannochloropsis gaditana*

**DOI:** 10.1186/s12934-022-01847-9

**Published:** 2022-06-16

**Authors:** Michela Cecchin, Stefano Cazzaniga, Flavio Martini, Stefania Paltrinieri, Simone Bossi, Massimo E. Maffei, Matteo Ballottari

**Affiliations:** 1 Dipartimento di Biotecnologie, Università degli Studi di Verona, Strada le Grazie 15, 37134 Verona, Italy; 2 Dipartimento di Scienze della Vita e Biologia dei Sistemi, Unità di Fisiologia Vegetale, Università di Torino, Via Quarello 15/a, 10135 Turin, Italy

**Keywords:** Astaxanthin, Microalgae, Omega-3 fatty acids, Eicosapentaenoic acid, Carotenoids

## Abstract

**Background:**

Astaxanthin is a ketocarotenoid with high antioxidant power used in different fields as healthcare, food/feed supplementation and as pigmenting agent in aquaculture. Primary producers of astaxanthin are some species of microalgae, unicellular photosynthetic organisms, as *Haematococcus lacustris*. Astaxanthin production by cultivation of *Haematococcus lacustris* is costly due to low biomass productivity, high risk of contamination and the requirement of downstream extraction processes, causing an extremely high price on the market. Some microalgae species are also primary producers of omega-3 fatty acids, essential nutrients for humans, being related to cardiovascular wellness, and required for visual and cognitive development. One of the main well-known producers of omega-3 fatty eicosapentaenoic acid (EPA) is the marine microalga *Nannochloropsis gaditana* (named also *Microchloropsis gaditana*): this species has been already approved by the Food and Drug Administration (FDA) for human consumption and it is characterized by a fast grow phenotype.

**Results:**

Here we obtained by chemical mutagenesis a *Nannochloropsis gaditana* mutant strain, called *S4*, characterized by increased carotenoid to chlorophyll ratio. *S4* strain showed improved photosynthetic activity, increased lipid productivity and increased ketocarotenoids accumulation, producing not only canthaxanthin but also astaxanthin, usually found only in traces in the WT strain. Ketocarotenoids produced in *S4* strain were extractible in different organic solvents, with the highest efficiency observed upon microwaves pre-treatment followed by methanol extraction. By cultivation of *S4* strain at different irradiances it was possible to produce up to 1.3 and 5.2 mgL^−1^ day^−1^ of ketocarotenoids and EPA respectively, in a single cultivation phase, even in absence of stressing conditions. Genome sequencing of *S4* strain allowed to identify 199 single nucleotide polymorphisms (SNP): among the mutated genes, mutations in a carotenoid oxygenase gene and in a glutamate synthase gene could explain the different carotenoids content and the lower chlorophylls content, respectively.

**Conclusions:**

By chemical mutagenesis and selection of strain with increased carotenoids to chlorophyll ratio it was possible to isolate a new *Nannochloropsis gaditana* strain, called *S4* strain, characterized by increased lipids and ketocarotenoids accumulation. *S4* strain can thus be considered as novel platform for ketocarotenoids and EPA production for different industrial applications.

**Supplementary Information:**

The online version contains supplementary material available at 10.1186/s12934-022-01847-9.

## Background

*Nannochloropsis gaditana* (named also *Microchloropsis gaditana*) is a microalgae species belonging to the *Eustigmatophytes* group which raised a high interest for industrial applications due to its fast growth rate and high lipids content. The latter can reach up to 70% of dry weight under stress conditions e.g., nitrogen deprivation [1, 2]. In addition, the fatty acids present in *Nannochloropsis* are usually composed of 35% polyunsaturated fatty acids (PUFA), including eicosapentaenoic acid (EPA, 20: 5ω3), an omega-3 fatty acid considered an important supplement for human diet [3]. Indeed, in humans, omega-3 fatty acids are required to maintain cell membranes, brain functions, and the transmission of nerve impulses under normal conditions and play a key role in the processes of transfer of oxygen to blood plasma, haemoglobin synthesis, and cell division [4, 5]. For these reasons, omega-3 fatty acids are recommended for the prevention and treatment of cardiovascular diseases and in neurological treatments, besides preventing degenerative brain diseases [6]. Omega-3 fatty acids are primarily produced by marine microalgae, however current production practices rely on extraction from fish or krill oils, because of lower production costs [4]. On the other hand, the commercialization of this fish oil-derived product is limited by the unpleasant taste and odor, the presence of carcinogenic contaminants, antibiotics, and heavy metals, as also by the stability issues of the oil resulting in high production costs [7, 8]. Moreover, the content of omega-3 in farmed fish essentially depends on the amount that the different species intake from their diet: marine fish oil and fishmeal are included in the diet of farmed fish to enhance their omega-3 fatty acid content [4]. Thus, paradoxically, the aquaculture sector is the major provider and user of omega-3 fatty acids, with associated sustainability and ethical issues. Algae omega-3 fatty acids are considered as possible alternative for omega-3 fatty acid supplementation for infant food fortification and for vegan and vegetarian consumption [9]. Accordingly, microalgae, and in particular the *Nannochloropsis* genus, are industrially promising candidates as EPA platform for human diet supplementation. An important feature of *Nannochloropsis* biomass is that it is approved by Food and Drug Administration (FDA) for human consumption (US Food and Drug Administration New Dietary Ingredient Notification Report #826, 2015), while in the case of EU there is already an application as a novel food for human consumption under Regulation (EC) No. 258/97.

However, currently, the use of *Nannochloropsis* to produce EPA is not available industrially due to the high costs associated with microalgae’s cultivation. Indeed, photosynthetic solar energy conversion efficiency of green algae does not exceed the 3% value, a much lower value than their theorical forecasted potential of 8–10% [10]. Microalgae have evolved to survive in habitats with sudden changes of light, low cell density and light-limiting conditions that reduce growth. These conditions are different compared to those found in photobioreactors (PBRs) where microalgal cells are cultivated in environments with high irradiance and high cell density. Selection of strains with interesting traits, random mutagenesis and genetic engineering are the tools nowadays available to improve performance of microalgae in PBRs. Several efforts have been already made to improve photosynthetic efficiency in microalgae: reducing of the light harvesting antenna complex to decrease the optical density in mass culture and improving resistance to photo-inhibition or enhancing carbon assimilation efficiency acting on RUBISCO activity, are some of the applied strategies [11]. Another approach to boost industrial revenues is to improve the quality of algal biomass either by increasing the production of high-value products or by introducing bioactive molecules by metabolic engineering [12].

Astaxanthin is a ketocarotenoid with a high anti-oxidant capacity [13] and proved to be safe for human consumption [14]. Thanks to its properties, astaxanthin has garnered huge commercial value in the last years and, together with β-carotene, representing more than half of the current carotenoid market [15]. The antioxidant activity of astaxanthin and, in general, of carotenoids is important for human health and carotenoid-rich diets protect from different diseases, such as cancer, cardiovascular diseases, arthritis, and can improve health in patients affected by AIDS, diabetes, macular degeneration and neurodegeneration [16]. Now, commercial astaxanthin production is realized by fermentation from yeasts and bacteria, by extraction from shrimp shells and microalgae, and through chemical synthesis from petrochemicals. Yeasts and bacteria producing astaxanthin are genetically engineered, thus their product cannot be considered for human consumption. Synthetic astaxanthin, that actually covers 95% of the market, is produced from petrochemical sources, raising issues of potential toxicity, pollution, and sustainability and posing severe health risks; hence chemically produced astaxanthin is sold in the animal feed market, but it does not meet the regulatory requirements to be used for direct human use in any country [15, 17]. This limitation is pushing the production of natural astaxanthin towards microalgal cultivation. Several microalgal species as *Haematococcus lacustris*, *Chromochloris zofingiensis* or *Chlamydomonas nivalis* are primary producers of astaxanthin [18]. Natural astaxanthin is indeed produced at industrial scale by cultivation of *H. lacustris* (previously known as *Haematococcus pluvialis*), where astaxanthin concentration up to 5% of its dry weight can be induced in stressing growth conditions as nutrient starvation, high light, high salinity or high/low temperature [19]. Indeed, astaxanthin production by *H. lacustris* requires a two-step cultivation system: in the first step “green” biomass is generated, while in the second step “red” astaxanthin biosynthesis is induced by stressing the cell cultures that form cysts [20]. The rigid cell wall of this microalgal species, composed of a trilaminar sheet, requires costly disruptive methods for its degradation and negatively affects yield, quality, and bioavailability of the recovered bioactive compounds. Thus, *H. lacustris* derived astaxanthin corresponds to  < 1% of the commercialized astaxanthin quantity [20]. On the other hand, synthetic astaxanthin presents much lower antioxidant properties compared to the natural one [20], and it has not been approved for human consumption by FDA. Alternative platforms have been attempted to overcome such limitations, from plants to different species of microalgae, but a commercially viable system has yet to be made [12]. In *N. gaditana* violaxanthin, β-carotene and vaucheriaxanthin esters are the major accumulated carotenoid, while zeaxanthin and ketocarotenoids as canthaxanthin and astaxanthin are present to a lower extent [21, 22].

This work was focused to isolate mutant strains of *N. gaditana* with an improved content of carotenoids interesting for industrial application. A library of *N. gaditana* mutant strains was produced by chemical mutagenesis and screened for a higher carotenoids-to-chlorophylls ratio. Surprisingly, among the mutant strains generated, the strain called *S4* accumulated a significant amount of astaxanthin. Moreover, the total lipid content was increased in the mutant in absence of nitrogen starvation, suggesting *S4* strain as a potential candidate for industrial production of both astaxanthin and EPA. *S4* genome was also sequenced allowing to correlate the observed phenotype with the genotype.

## Results

### Mutagenesis and mutant selection

**Fig. 1:**
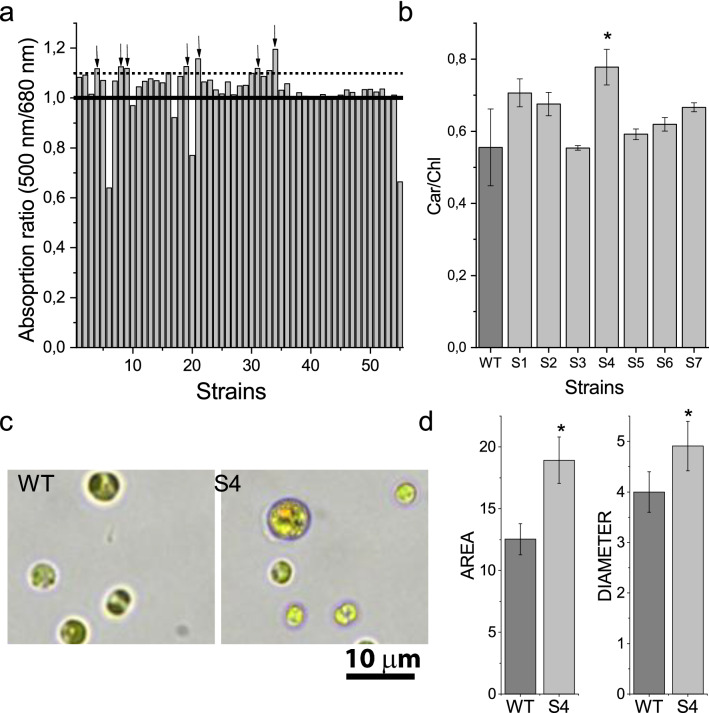
Screening of *Nannochloropsis gaditana* strains with increased carotenoids per chlorophyll content. **a** 500/680 nm absorption ratio of strains previoulsy selected for having a visible orange phenotype. The 500/680 nm absoprtion ratio meaured were normalized as a percentage to average 500/680 nm value calculated considering all strains analyzed. The strains with 500/680 nm absorption ratio increased by at least 10% compared to the average were further analyzed and are indicated with arrows. **b** Carotenoids per chlorophylls content of selected mutant strains compared to WT analyzed by HPLC. **c** Microscopy images of WT and *S4* cells. **d** Average diameter and area of WT and *S4* strains Error bars indicate standard deviation (n = 3 for panel b, n = 75 for panel **d**. Significant different values are marked with an asterisk as determined by unpaired two-sample t-test (p < 0.05)

*N. gaditana* mutant strains were generated by random mutagenesis of WT strain by the alkylating agent ethyl methane sulfonate (EMS) as previously reported [23, 24]. EMS induces single-point mutations (SNPs) by nucleotide substitution, particularly by guanine alkylation. The survived colonies were screened for a visible altered color in plates. Selected colonies were grown in liquid medium and the carotenoid/chlorophyll ratio was investigated by analyzing the 500/680 nm absorption ratio in 96-wells microtiter: 500 nm and 680 nm are indeed wavelengths where essentially only carotenoids and chlorophyll *a* absorb, respectively, and the 500/680 absorption ratio can be used as an indication of the Car/Chl ratio (Fig. 1a). Colonies which resulting value was increased at least by 10%, compared to the average of the other colonies, were further analyzed upon cultivation in 50 mL flasks. Pigments were extracted and analyzed by HPLC, to precisely determine the Car/Chl ratio (Fig. 1b). Only one mutant showed a significant increase compared to WT: this mutant, called *S4*, presented a  ~ 40% higher Car/Chl ratio with respect to WT. Interestingly, S4 cells were characterized by larger cell diameter and area compared to WT (Fig. 1c, d).

### Pigment composition of *S4* mutant strain

Carotenoids and chlorophylls content per cells of WT and *S4* mutant strain were analyzed by HPLC (Table 1). *S4* strain was characterized by a  ~ 50% reduction of total chlorophyll *a* content per cell. Carotenoids content per cell was also reduced, but to a lower extent, because of the increased Car/Chl ratio observed in this strain compared to the WT. In the *S4* mutant, on a chlorophyll basis, the increase in total carotenoids content was related to an increase in violaxanthin and, surprisingly to a significant high content of astaxanthin and canthaxanthin. Both astaxanthin and canthaxanthin were previously reported in *N. gaditana* reaching values of 3–4% of total carotenoids, with astaxanthin accumulating in the WT at a 1.4 ± 0.6% of total carotenoids and canthaxanthin 10.1 ± 3.2% [22]. In the *S4* mutant, astaxanthin and canthaxanthin represented respectively 14.4 ± 4.5% and 14.7 ± 1.6% of total carotenoid. The increase in ketocarotenoids content in the *S4* strain was accompanied to a decreased in β-carotene, both on a chlorophyll basis and as percentage of total carotenoids produced. The percentage of zeaxanthin and vaucheriaxanthin on total carotenoids were also reduced in *S4* strain compared to WT. It is important to note that β-carotene and zeaxanthin are indeed the main substrates for both ketocarotenoids and vaucheriaxanthin biosynthesis [25, 26].

**Table 1:** Pigment analysis of WT and *S4* mutant strain

	Chl/cell (%)	Chl *a* (pmol)	Vio (pmol)	Asta (pmol)	Vau (pmol)	Zea (pmol)	Cantha (pmol)	β-car (pmol)
WT	100.0	100	23.74	0.69	12.49	3.60	5.16	5.35
sd	5.6		6.14	0.30	2.06	0.25	1.51	0.45
*S4*	51.4**	100	36.88*	11.20*	11.42	3.28	11.41**	3.61**
sd	5.5		1.88	3.43	0.47	0.28	1.06	0.38

	Car/cell (%)	Car/Chl	Vio (%Car)	Asta (%Car)	Vau (%Car)	Zea (%Car)	Cantha (%Car)	β-car (%Car)
WT	100.0	0.51	46.5	1.4	24.5	7.1	10.1	10.5
sd	20.0	0.10	13.5	0.6	5.1	1.0	3.2	1.6
*S4*	75.3	0.78*	47.4	14.4*	14.7*	4.2*	14.7	4.6**
sd	9.3	0.05	3.5	4.5	1.0	0.4	1.6	0.6

Chlorophyll content per cell (Chl/cell) was set to 100% in the case of WT. The concentration of pigments in pmol was determined by HPLC and normalized to 100 pmol of chlorophyll a (Chl). Errors are reported as standard deviations (sd) and significantly different values are marked with * if p < 0.05 or ** if p < 0.01, as determined by unpaired two-sample t-test (n = 3)

*Asta* Violaxanthin: Vio astaxanthin, *Vau* vaucheriaxanthin, *β-Car* β-carotene, *Zea* zeaxanthin, *Cantha* canthaxanthin

### Physiologic characterization of *S4* strain

To investigate the possible physiologic effects of the altered pigment composition of the *S4* mutant as compared to WT, different photosynthetic parameters based on fluorescence measurement were investigated. F_v_/F_m_ parameter, representing the Photosystem II (PSII) maximum quantum efficiency was 0.678 ± 0.004 and 0.655 ± 0.016 in WT and *S4* mutant respectively, suggesting that the increased Car/Chl ratio of the mutant has a minor effect on PSII quantum efficiency. Light harvesting properties of PSII were thus analysed by measuring chlorophyll *a* fluorescence induction kinetics in DCMU-treated cells. DCMU is an inhibitor of PSII, and, in these conditions, the chlorophyll *a* fluorescence emission is not influenced by PSII photochemical activity: in limiting light condition, the kinetic of chlorophyll fluorescence emission of DCMU-treated cells is thus related to the light harvesting capacity of PSII. In particular, the reciprocal of the time required to reach 2/3 of the maximum fluorescence emission (1/τ_2/3_) has been usually adopted to evaluate the PSII light harvesting capacity, called also PSII antenna size [27]. No difference in chlorophyll *a* fluorescence induction kinetics were detected in *S4* compared to WT (see Additional file 1: Figure S1): this suggests that changes in the pigments content did not affect the PSII light harvesting capacity.

**Fig. 2:**
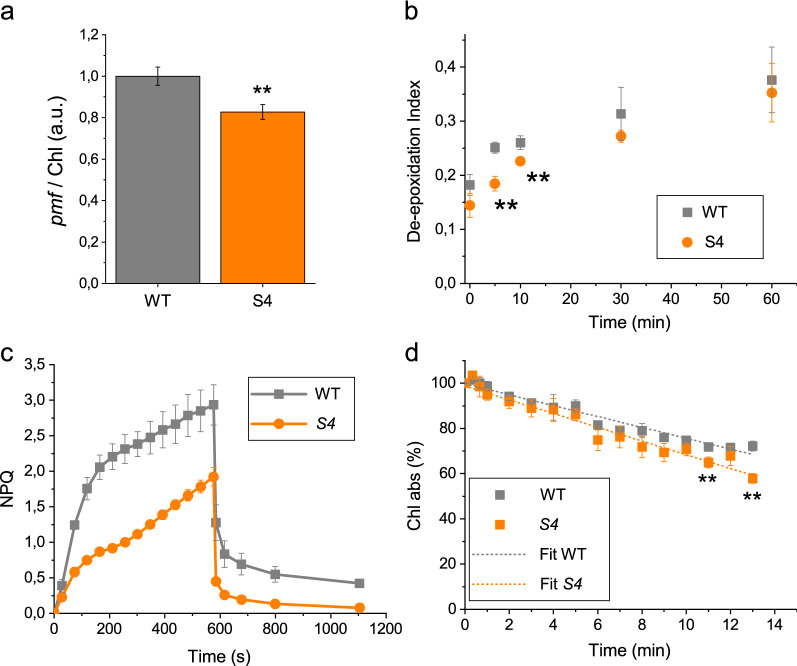
ECS, de-epoxidation index, NPQ and chlorophylls bleaching of S4 strain compared to WT. **a** Proton motive force (*pmf*) obtained by electrochromic shift measurement (ECS) on whole cells at 1000 µmol photons m^−2^ s^−1^ and normalized to chlorophylls content. **b** De-epoxidation index of WT and *S4* strains upon exposure to high light (2500 µmol photons m^−2^ s^−1^) for 1 h. Pigment composition was evaluated at different time points by HPLC analysis and de-epoxidation index was calculated as (zeaxanthin + antheraxanthin/2) /(antheraxanthin + violaxanthin + zeaxanthin). **c** Non-photochemical quenching (NPQ) induction of WT and *S4* mutant strain at actinic light of 1500 µmol photons m^−2^ s^−1^. After 10 min of illumination actinic light was turned off to induce NPQ relaxation. **d** Chlorophylls bleaching kinetics: absorption of chlorophylls was measured for about 14 h upon exposure of cells to 2500 µmol photons m^−2^ s^−1^. Experimental data were fitted with linear function. Error bars are reported as standard deviation (n = 3). Significant different values are marked with ** as determined by unpaired two-sample t-test (n = 3, p < 0.01)

Light dependent proton transport across thylakoid was then estimated by measuring the electrochromic shift (ECS) of carotenoid absorption upon exposure to light [28]. ECS signal is indeed related to a shift of carotenoid absorption because of the electrochemical gradient induced by photosynthetic proton transport. As reported in Fig. 2a, on a chlorophyll basis, a significant decreased of light-dependent proton motive force (*pmf*) was observed in *S4* compared to WT.

Lumen acidification is the trigger of zeaxanthin biosynthesis, due to activation of the violaxanthin de-epoxidase (VDE) enzyme which convert violaxanthin to zeaxanthin. Light dependent zeaxanthin accumulation was thus investigated in the WT and mutant strain upon exposure to strong light (2500 μmol photons m^−2^ s^−1^) for 1 h. As reported in Fig. 2b, in the *S4* mutant the de-epoxidation index, representing the capacity of VDE to convert violaxanthin to zeaxanthin, was reduced in the first 10 min of the experiment, in line with the reduced *pmf* observed. On a longer timescale, zeaxanthin accumulation was similar in *S4* and WT strains.

In the *S4* strain, the altered pigment composition, reduced *pmf* and delayed violaxanthin de-epoxidation could potentially influence its photoprotective properties. Non-photochemical Quenching (NPQ) is an important photoprotective mechanism that dissipates the excess of absorbed light as heat: in *N. gaditana* it was previously reported that NPQ is triggered by lumen acidification and that it depends on the conversion of the violaxanthin to zeaxanthin [29]. NPQ induction kinetics were thus evaluated in *S4* and WT cells (Fig. 2c), observing a  ~ 30% reduced NPQ in the *S4* strain. Since NPQ induction is one of the major photoprotective mechanisms observed in photosynthetic organisms, the photosensitivity of *S4* compared to WT was investigated by measuring the photobleaching kinetics upon exposure to a strong light (2500 μmol photons m^−2^ s^−1^) for several hours. Chlorophylls bleaching was measured upon pigment extraction and measurement of chlorophyll absorption in the 600–750 nm spectral region. Chlorophylls absorption decreased by  ~ 30% after 14 h of illumination in the WT strain, whereas the *S4* mutant strain was characterized by a faster photobleaching kinetics (Fig. 2d). This result demonstrates that the reduction in NPQ induction causes a slightly higher photosensitivity in the *S4* mutant.

**Fig. 3:**
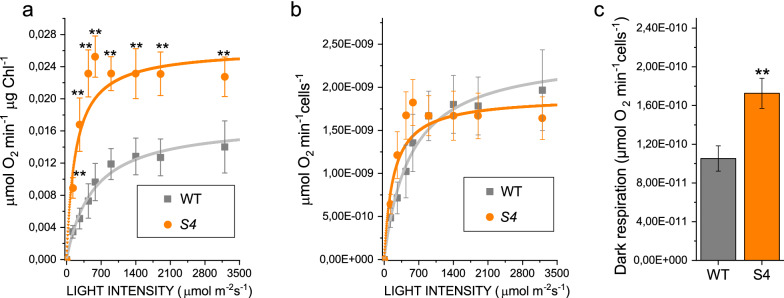
**a** Net oxygen evolution rate at different light intensities for WT and *S4* mutant strains normalized on a chlorophyll basis. Experimental data were fitted with hyperbolic function. **b** Net oxygen evolution rate normalized on a cell basis. **c** Dark respiration rate normalized on a cell basis for WT and *S4* mutant strains. Error bars showed the standard deviation (n = 3). Significant different values are marked with ** as determined by unpaired two-sample t-test (n = 3, p < 0.01)

Photosynthetic performances were then evaluated by calculating the light dependent oxygen evolution curve (Fig. 3). On a chlorophyll basis, the net oxygen evolution curves were characterized by a higher production rate in *S4*. As reported in Fig. 3a, the P_max_, being the maximum oxygen evolution rate value, was almost doubled in *S4* when compared to WT. This result is consistent with previous data obtained in a *N. gaditana* mutant strain with a similar 50% reduction in chlorophyll content per cell [24]. It is interesting to note that when oxygen evolution traces were normalized on a cell basis, despite the reduction of chlorophyll content in *S4* strain, the P_max_ measured were not significantly affected (Fig. 3b). A 70% increase in dark respiration was also measured in the *S4* strain, compared to the WT (Fig. 3c). Suggesting an increased mitochondrial activity in the *S4* strain.

Increased light dependent oxygen production on a chlorophyll basis suggests that electron transport is even increased in the *S4* strain, compared to WT, suggesting that the reduced *pmf* observed in the *S4* strain is not related to an impaired photosynthetic electron transport.

### Biomass yield and lipid productivity of *S4* strain

**Fig. 4:**
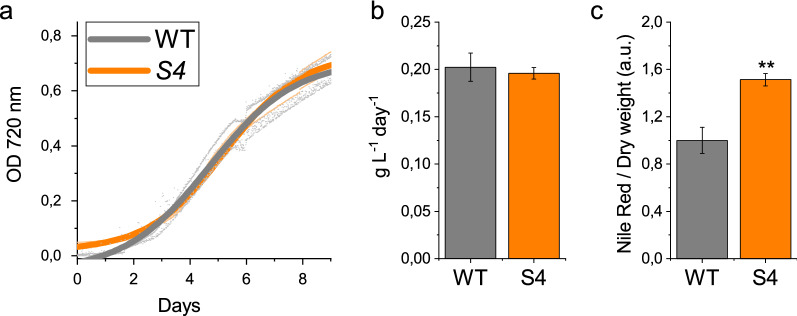
Growth curve, biomass, and lipid productivity of WT and *S4* mutant strain. **a** Growth curve obtained measuring absorption at 720 nm. **b** Daily biomass productivity expressed as g L^−1^ day^−1^. **c** Lipid accumulation per dry weight measured by Nile red staining in cells at the end of the growth curve. Error bars are reported as the standard deviation (n = 3). Significant different values are marked with ** as determined by unpaired two-sample t-test (n = 3, p < 0.01)

Biomass productivity was evaluated in the *S4* mutant strain as compared to WT in airlift PBRs under continuous white light at 400 μmol photons m^−2^ s^−1^. This light intensity was previously reported to induce light saturation in *N. gaditana* exposed to direct illumination [24]. As reported in Fig. 4a and b, growth kinetics and biomass productivity were similar between *S4* and WT strains. The reduction of NPQ and the slight increase in photosensitivity of the mutant compared to WT (Fig. 2) did not compromise growth at the irradiances conditions herein tested. Lipid content was evaluated at the end of the growth curve by Nile red staining. In the *S4* mutant strain an increased Nile red fluorescence per dry weight was observed (+ 87%, Fig. 4c) when compared to WT, suggesting an increased lipid fraction in the mutant strain with respect to the WT. These results suggest that the increased photosynthetic activity measured in the *S4* strain (Fig. 3) rather than causing an increased biomass accumulation, allows to increase the production of biomass component with a high chemical energy associated, such as lipids. Being lipids the macromolecules with the associated highest energy density, an increased lipid fraction is an indication of a better light use efficiency: this could be related to the reduced NPQ induction, dissipating less energy as heat, and to the reduced Chl/Cell ratio that allows a better light distribution in the PBR.

Upon exposure to nitrogen deficiency, *N. gaditana* is known to boost lipid accumulation with the carbon flow redirected toward nitrogen-free lipids molecules [1, 30]. To evaluate the effect of nitrogen starvation on the *S4* mutant, cells at stationary phase were harvested and moved to a nitrogen depleted F/2 medium for other 7 days to induce nitrogen starvation. In the WT strain the absence of nitrogen induced a 45% increase of lipid content per dry weight compared to the nitrogen replete condition, whereas in the *S4* mutant this increase was not observed (see Additional file 1: Figure S2). Moreover, in the *S4* strain, astaxanthin and ketocarotenoids content per dry weight was similar in nitrogen deficiency compared to nitrogen replete conditions (see Additional file 1: Figure S3), suggesting that in the *S4* strain a stress phase is not required to boost astaxanthin accumulation.

### Feasibility of *S4* strain pigments extraction

The cell wall of *N. gaditana* is formed by two layers, a cellulosic internal one and an external hydrophobic layer composed of alginates [31]. To evaluate the feasibility of carotenoid extraction from the *S4* strain, different extraction methods were evaluated. As reported in Table 2, different solvents were applied to algal biomass with or without pre-treatments to break or weaken the cell wall. The pre-treatments applied were high-pressure homogenizer, microwaves, or sonication. After treatments, the samples were lyophilized and then resuspended in the various solvents. Pigments extraction efficiencies were estimated considering DMSO extraction as control because this solvent was extracting all the pigments in *Nannochloropsis* cells regardless of cell pre-treatments. As reported in Table 2, in the absence of cell pre-treatments, up to  ~ 38% of total carotenoids were extracted using methanol or ethanol as solvent, while extraction efficiency was reduced to  ~ 14% in the case of mineral oil or in soybean oil. Cell pre-treatments increased the extraction efficiency, with the highest yield in the case of high-pressure homogenizer treatment, which allows to extract essentially the carotenoids from algal biomass using methanol or acetone as solvents, followed by ethanol (79% of carotenoids extracted), ethyl-acetate (75%) and oil extraction (39.3% and 20.6% respectively for mineral oil and soybean oil). Sonication and microwave pre-treatment improved the extraction efficiency when compared to untreated samples but with lower extraction efficiencies compared to high-pressure homogenizer treatment (Table 2). Again, methanol was the best solvent for pre-treated cells with sonication and microwaves, yielding extraction efficiency of  ~ 80% for both pre-treatments.

**Table 2:** Carotenoid extraction efficiency in different solvents

Solvent	No pre-treatment (%)	High-pressure homogenizer (%)	Sonication (%)	Microwaves (%)
DMSO	100.0	100.0	100.0	100
Acetone	35.8	100.0	49.2	37.9
Ethyl-acetate	29.0	75.8	40.6	32.2
Methanol	38.1	98.1	85.4	79.2
Ethanol	37.7	79.3	54.2	69.5
Mineral oil	13.5	39.3	14.3	14.6
Soybean oil	14.0	20.6	16.4	16.0

Carotenoid extraction efficiency were estimated for the different solvents by comparing the absorption spectra of the extracts with the absorption spectrum of DMSO extracts, used as control. Carotenoid contribution to pigments absorption were determined by absorption spectrum deconvolution with carotenoids and chlorophyll a absorption forms

### Optimization of ketocarotenoids and EPA accumulation in *S4*

The capacity of *S4* to accumulate astaxanthin makes this strain interesting for potential industrial applications. To identify the optimal conditions for astaxanthin production, the *S4* strain was grown at different light intensities (100–500–1000–2000 µmol photons m^−2^ s^−1^) to evaluate their influence on ketocarotenoids accumulation. These experiments were done by fluxing air enriched with 3% CO_2_ in the PBR to avoid possible carbon limitations. In parallel with autotrophic growth, the *S4* strain was also cultivated in mixotrophy by using 1% glucose to boost biomass and carotenoid productivity, as recently reported for *N. gaditana* strains grown outdoor [32]. When cells reached the stationary phase, the culture was diluted 1:10 with fresh medium for a second cycle of growth. At the end of this second cycle, the biomass was harvested and analysed.

As reported in Table 3, the highest biomass productivity was observed at 500 µmol m^−2^ s^−1^. The addition of glucose did not significantly influence cell growth, consistently with recent findings in this species [33]. The fraction of ketocarotenoids (Table 3) and astaxanthin per dry weight were similar in all conditions tested, with the exception of the highest irradiance used (2000 µmol m^−2^ s^−1^), where these values were reduced. Consequently, the highest volumetric productivities of ketocarotenoids and astaxanthin were observed in correspondence of growth conditions inducing the highest biomass productivity at irradiances of 500 µmol m^−2^ s^−1^ either in the presence or absence of glucose (Fig. 6a).

**Table 3:** Biomass, EPA and ketocarotenoids productivity

	Biomass productivity (g DW L^−1^ day^−1^)	Ketocarotenoids (mg g DW^−1^)	Astaxanthin (mg g DW^−1^)	Ketocarotenoids (mg L^−1^ day^−1^)	EPA (mg g DW^−1^)	EPA (mg L^−1^ day^−1^)
100 µmol m^−2^ s^−1^	0.23 ± 0.02^a^	3.33 ± 0.35^a^	1.78 ± 0.19^a.b^	0.77 ± 0.10^a^	13.13 ± 1.52^a.b^	3.02 ± 0.44^a^
100 µmol m^−2^ s^−1^ + 10 gL^−1^ Glu	0.25 ± 0.05^a.b^	3.54 ± 0.33^a^	1.80 ± 0.23^a.b^	0.89 ± 0.20^a.b^	18.48 ± 2.04^a^	4.62 ± 1.06^a^
500 µmol m^−2^ s^−1^	0.35 ± 0.01^c^	3.72 ± 0.12^a^	1.89 ± 0.15^a^	1.30 ± 0.06^c^	12.36 ± 2.61^b^	4.33 ± 0.92^a^
500 µmol m^−2^ s^−1^ + 10 g L^−1^Glu	0.39 ± 0.06 ^c^	3.61 ± 0.19^a^	1.73 ± 0.26^a.b^	1.41 ± 0.23^c^	13.38 ± 4.70^a.b^	5.22 ± 2.00^a^
1000 µmol m^−2^ s^−1^	0.26 ± 0.01^a.b^	4.05 ± 0.13^a^	2.19 ± 0.07^a^	1.05 ± 0.05^a.c^	16.05 ± 3.32^a.b^	4.17 ± 0.88^a^
1000 µmol m^−2^ s^−1^ + 10 g L^−1^Glu	0.32 ± 0.05^b.c^	3.97 ± 0.91^a^	2.19 ± 0.50^a^	1.27 ± 0.35^b.c^	12.57 ± 0.96^a.b^	4.02 ± 0.70^a^
2000 µmol m^−2^ s^−1^	0.31 ± 0.01^a.b.c^	2.12 ± 0.05^b^	1.10 ± 0.03^c^	0.66 ± 0.03^a^	11.37 ± 1.14^b^	3.53 ± 0.37^a^
2000 µmol m^−2^ s^−1^ + 10 g L^−1^Glu	0.36 ± 0.03^c^	2.31 ± 0.04^b^	1.25 ± 0.03^b.c^	0.83 ± 0.07^a^	12.60 ± 2.02^a.b^	4.54 ± 0.82^a^

Biomass, EPA and ketocarotenoids productivity of *S4* cells grown at different irradiances with or without glucose (Glu). Standard deviations are reported (n = 4). Statistical analysis of data was performed using the one-way analysis of variance (ANOVA) followed by a post hoc Tukey’s test. Letters denote statistically significant variations (p < 0.05)

**Fig. 5:**
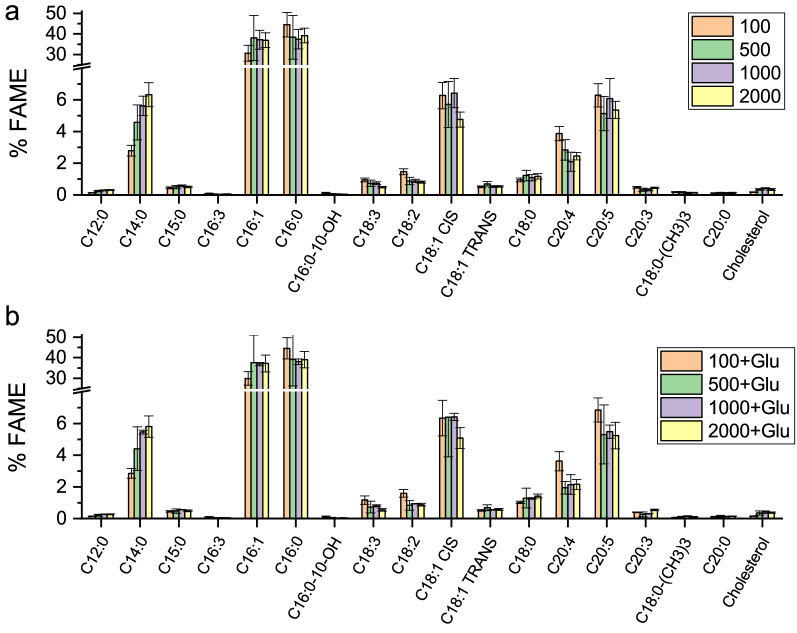
FAME composition of *N. gaditana S4* strain. FAME composition of *S4* strain is reported as percentage of total FAME for cells grown in absence (**a**) or presence (**b**) of 10% glucose (Glu) in the growth medium. The different irradiances of growth, from 100 to 2000 µmol m^−2^ s^−1^, are reported

**Fig. 6:**
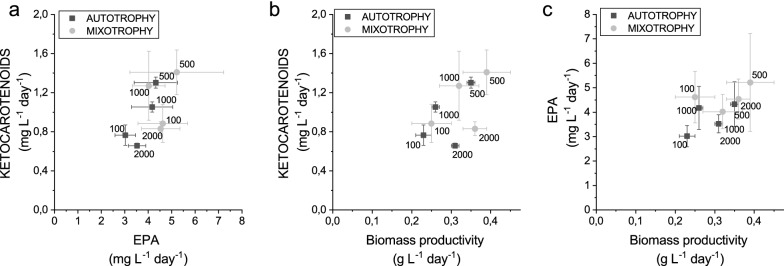
Correlation between EPA and ketocarotenoids content. The dark squares refer to a growth medium without glucose, instead grey dot refer to a growth medium with 10 g L^−1^ of glucose. Error bars showed the standard deviation (n = 4)

Considering the potential of *N. gaditana* as a bio producer of fatty acid omega-3 as EPA, lipid content and composition were analysed at the different growth conditions herein evaluated. In particular, the lipid fraction of the *S4* mutant strain was analyzed by GC analysis of derivatized Fatty acid methyl esters (FAME). The percentage of FAME per dry biomass produced was not significantly affected by the different growth cultivation conditions herein applied (see Additional file 1: Table S1). Palmitic acid (C16:0) and palmitoleic acid (C16:1) were the major fatty acids accumulated, followed by myristic acid (C14:0), oleic acid (cis C18:1), EPA (C20:5) and arachidonic acid (C20:4) (Fig. 5). FAME composition was similar in autotrophic and mixotrophic growth conditions. At the irradiance where the highest biomass productivity was observed (500 µmol m^−2^ s^−1^) FAME composition of S4 strain was compared to the WT case observing a similar FAME composition with only minor changes as reduced accumulation in *S4* of pentadecanoic acid (C15:0), linoleic acid (C18:2) and stearic acid (C18:0) while arachidonic acid (C20:4) was increased (see Additional file 1: Figure S4). Interestingly, traces of lauric acid (C12:0), 10-hydroxy hexadecanoic acid (C16:0), hexadecatrienoic acid (C16:3), trimethoxy-octadecanoic acid (C18:0), γ-linolenic acid (C18:3), eicosatrienoic acid (C20:3) arachidic acid (C20:0) and cholesterol were found only in *S4* mutant but not in WT cells, likely because of the altered lipid metabolism in the mutant. In *S4* strain, the main influence of light intensity was an increase in myristic acid (C14:0) and a decrease in arachidonic acid (C20:4) at increasing light intensities in both autotrophic and mixotrophic growth (Fig. 5). In the case of EPA, the highest fraction on total FAME was observed at 100 µmol m^−2^ s^−1^ (Fig. 5), but the fraction of EPA per dry weight were similar in the different conditions. EPA volumetric productivities were not significantly affected by the different growth conditions obtained in the different conditions (Table 3, Fig. 6b). Interesting we observed that there was no clear correlation between EPA and ketocarotenoids production (Fig. 6c), indicating that the simultaneous production of both EPA and ketocarotenoids was not affected by a metabolic competition. The highest EPA and ketocarotenoids volumetric productivity could be obtained at 500 µmol photons m^−2^ s^−1^, either in presence or absence of glucose.

### *S4* genome sequencing

To investigate the possible genetic traits at the base of the *S4* genotype induced by EMS mutagenesis, the genome of the mutant strain was sequenced and compared with its background. WT and *S4* genomes were sequenced and compared with the reference genome available for *N. gaditana* [34]. A mapped coverage of 115X for WT and 84X for *S4* was obtained (see Additional file 1: Table S2). The *S4* genotype revealed the presence of 199 proprietary mutations affecting 195 genes (see Additional file 1: Table S3 and Additional file 2). To summarize the overall effect of the introduced SNPs, Gene Ontology (GO) terms were used: several biological processes, molecular functions and cellular component were potentially affected by mutations (see Additional file 1: Figure S5). Sixty-six genes presented mutations potentially affecting the encoded protein activity. Six genes had mutations potentially affecting translation of the protein (see Additional file 1: Table S4), in particular they comprised: two hypothetical proteins (Naga_100022g21 and Naga_100016g35), a GTPase (Naga_100047g12), a mediator of RNA polymerase II transcription (Naga_100062g8), a ccr4-associated factor (Naga_100037g9) and a e3 ubiquitin-protein ligase (Naga_100021g66). The proteins encoded by these mutated genes are involved in expression or signal transduction: it is thus difficult to draw a correlation between these mutations and the observed phenotype of the *S4* strain. Considering the peculiar decrease in chlorophyll content and increased astaxanthin production of the *S4* mutant, SNPs on genes encoding proteins predicted to be targeted to the chloroplast were then considered (see Additional file 1: Table S5): four genes were identified with SNPs inducing a missense variant. Among them a carotenoid oxygenase (Naga_100050g23) was found with a substitution of a glycine in the position 720 with a serine. Naga_100050g23 gene was also reported to be upregulated upon exposure to high light [35]. Carotenoid cleavage oxygenase family proteins catalyse the oxidative cleavage of carbon–carbon double bonds in carotenoid backbones [36], therefore the mutation in Naga_100050g23 gene could be responsible for the altered carotenoid biosynthetic pathway observed in *S4* mutant. It is interesting to note that no beta-carotene ketolase (BKT) enzyme has been identified in *N. gaditana* genome. Considering that even in the WT canthaxanthin and traces of astaxanthin could be retrieved, the molecular details at the base of biosynthetic pathway of these ketocarotenoids requires to be elucidated.

Another mutated gene in the *S4* strain, which protein was predicted to be targeted to chloroplast, is a glutamate synthase (Naga_100005g23) where a missense mutation leads to a substitution of a proline with a serine, causing a possible reduced activity for this enzyme involved in nitrogen assimilation. Nitrogen metabolism is fundamental for chlorophyll biosynthesis and lipid metabolism; therefore, a mutation on a glutamate synthase is a candidate to explain the reduced chlorophyll content per cell and the increased lipid accumulation phenotype observed in the *S4* mutant. Eukaryotic glutamate synthase (GS) enzyme catalyses the formation of glutamine from glutamate and ammonia with the hydrolysis of ATP.GS1 isoforms are localized in cytosol and GS2 isoforms in plastid. In *A. thaliana* it has been previously reported that GS2 is involved in nitrogen use efficiency and biomass accumulation, being a central regulator between the nitrogen and the carbon cycles, moreover GS2 plays a key role in plant responses to abiotic stress [37]. Indeed, GS2 knock-out mutant of *A. thaliana* showed dwarf and chlorotic phenotype and an impaired nitrogen metabolism, however the mutant plants were able to grow and complete their life cycle under environmental air conditions and, surprisingly, the mutant plants were more tolerant to salt stress than WT. In the same mutants, genes encoding for cytosolic GS1 isoforms were found overexpressed to compensate the lack of GS2 [37]. The central role of GS2 as a regulator of nitrogen and carbon metabolism were previously demonstrated also in wheat and rice plants [38, 39]. The similar biomass yield in the *S4* mutant compared to WT (on the contrary to *A. thaliana* GS2 mutants) could be related with the overexpression of cytosolic GS1 isoform or with the maintenance of partially protein activity of the plastid GS2 isoform.

## Discussion

Chemical mutagenesis and phenotypic screening of mutant libraries has been previously reported as an efficient method to confer new and specific properties to microalgal strains [23, 24, 40, 41]. *N. gaditana S4* mutant strain was selected for having an increased carotenoid content per chlorophylls. The increased Car/Chl ratio of *S4* strain was due to reduced Chl content per cell: carotenoids content per cell was also reduced but to a lower extent. *S4* strain was further affected in pigment composition being characterized by an increased accumulation of both canthaxanthin and astaxanthin, the latter being found usually only in traces in *N. gaditana* [22]. The altered pigment composition did not change the light harvesting properties of PSII, but rather an increased photosynthetic activity was measured on a chlorophyll basis in *S4* strain (Fig. 3). This result is consistent with previous findings, where strains of *N. gaditana* with reduced chlorophyll content were characterized by improved photosynthetic activity and reduced thermal dissipation of the light energy absorbed [24]. Indeed, also in the case of *S4* strain, a strong reduction of NPQ was observed (Fig. 2C). The reduction of NPQ caused an increased, even if weakly, photosensitivity of the mutant compared to WT (Fig. 2D). However, this did not compromise growth that indeed was similar to the WT case (Fig. 4). Rather, increased lipid content was measured in *S4* strain compared to WT. It is important to note that *S4* cells were in average larger than WT cells (Fig. 1c, d), a feature which could possibly be related to increased lipid content per cells (see Additional file 1: Figure S2). Being lipids the macromolecules with the highest energy density an increased lipid content is an indication of a better light use efficiency: this could be related to the reduced NPQ induction, thus dissipating less energy as heat, and to the reduced Chl/Cell ratio allowing for a better light distribution in the case of *S4* strain cultivation. FAME composition was similar for S4 and WT cells with only some minor alteration in fatty acids representing in total  ~ 10% of the overall FAMEs in the two strains (see Additional file 1: Figure S4). Among the different fatty acid accumulated in *N. gaditana*, EPA is of particular interest being required for human health [4, 42, 43].

By growing the *S4* strain at different irradiances, from 100 to 2000 µmol photons m^−2^ s^−1^ it was possible to produce ketocarotenoids and EPA in the range respectively of 0.77–1.41 and 11.37–18.48 g L^−1^ day^−1^. Positive correlation between EPA and ketocarotenoids accumulation was observed (Fig. 6), suggesting that no direct metabolic competition occurs for the biosynthesis of these metabolites in *N. gaditana S4* strain. It is important to note the ketocarotenoids produced are composed almost equally by astaxanthin and canthaxanthin, being both relevant as antioxidants for nutraceuticals, feed, and cosmetic applications. Canthaxanthin is indeed used as a skin-tanning agent in cosmetics, and it was reported to be capable of scavenging reactive oxygen species and quenching singlet oxygen [44]. Moreover, canthaxanthin supplementation enhanced the antioxidant defence in rat livers leading to reduced oxidative damage of DNA [45, 46].

The ketocarotenoids produced in *S4* strains could be extracted in different solvent, with the best results obtained in the case of methanol or acetone extraction upon cell pre-treatment with a high-pressure homogenizer (Table 2). Other cells pre-treatments as sonication and microwaves pre-treatments improved the pigment extraction efficiency, but with lower efficiencies compared to high pressure homogenizer pre-treatment (Table 2). However, it is also essential to consider the energy costs associated with the individual treatments: sonication was suggested to have a cost in the range between 132 and 360 MJ/kg of biomass, depending on a multitude of factors such as the type of algae treated, temperature and reaction time and the presence or absence of microbeads in solution [47]. Microwaves and high-pressure homogenization were reported to have a cost of 420 and 529 MJ/kg of dried biomass, respectively. Methanol or ethanol extractions upon microwaves pre-treatment is thus likely the best compromise in terms of extraction efficiency and cost of pre-treatment. Other pre-treatments as enzymatic digestion of cell wall and/or mechanical treatment could be further evaluated. However, it is important to note that 75–100% of carotenoids accumulated in the *S4* strain could be extracted in organic solvent without incurring in the use of costly methods as supercritical CO_2_ extraction, as normally done in the case of astaxanthin extraction from *H. lacustris.*

The *S4* strain can thus be considered for the conversion of CO_2_ into organic biomass enriched in ketocarotenoids and EPA, with different potential industrial applications. Moreover, *N. gaditana* is a marine alga thus its growth in saltwater allows to mitigate the risk of contamination due to the selective pressure of the high salinity of the growth medium, paving the way for large scale application. It is important to note that even if astaxanthin percentage per dry weight was reduced when compared to *H. lacustris*, where up to 5% astaxanthin can be accumulated, in the *S4* strain astaxanthin accumulation does not require a stress phase*,* and a single cultivation stage can be adopted to produce a biomass enriched in both EPA and ketocarotenoids.

The identification of the genetic basis for the observed phenotype of *S4* strain is challenging. The genome sequencing allowed to identify 199 SNPs, among them a mutation in a carotenoid oxygenase gene and in a glutamate synthase gene could explain the different carotenoids content and the lower chlorophylls content, respectively. RNA-seq analysis could confirm our hypothesis and will help us to understand the general reorganization of genes expression and regulation in the mutant. Indeed, one disadvantages of chemical mutagenesis is the high number of induced mutations: several mutations affected components of signal transduction and the regulation of transcription and protein machinery; therefore, the final effect on the observed phenotype is difficult to predict due to the wide range of action of these proteins. Additional studies are under way for the identification of the mutation responsible of the phenotype: studies with CRISPR technologies will allow to evaluate the contribution of each mutation and will be interesting to further increased production of high value product as astaxanthin and EPA in *N. gaditana* and in other algae of interest.

## Conclusions

*Nannochloropsis gaditana* has been recently proposed and approved by FDA as a novel food for human consumption. The interest in this species relies on its fast growth phenotype, high lipid content and production of omega-3 fatty acids, including EPA. The *N. gaditana S4* strain was selected for having an increase carotenoid to chlorophyll ratio and resulted to accumulate high levels of both astaxanthin and canthaxanthin, in addition to EPA at different irradiances in a single cultivation phase.

Ketocarotenoids production by *S4* cultivation allow to avoid the so-called stress (or red) stage, required in the case of the latter to boost astaxanthin accumulation in the microalgae species currently used for astaxanthin production at industrial level, *H. lacustris.* Moreover, the possibility of *S4* strain to grown in saline environment permits to create a selective growth medium mitigating the risk of contamination. *S4* mutant strain could thus be utilized in aquaculture as fish feed or as novel food for human consumption to provide antioxidants and EPA. Further experiments are under way to optimize and scale-up the cultivation of the *S4* strain which can be considered as an innovative solution for producing astaxanthin, canthaxanthin and EPA.

## Methods

### Growth conditions and mutant generation

*Nannochloropsis gaditana* strain 849/5 from CCAP culture collection was used as WT strain for the generation of the mutant strain *S4* and for the comparison with *S4* during its characterization. For the screening procedure and physiological characterization, cells were grown under a 16/8 h light/dark photoperiod in low light (LL) condition of about 70 µmol photons m^−2^ s^−1^. Cells were grown in F/2 medium and chemical mutagenesis was performed as described in [23]. The survived colonies were screened on the base of visible altered pigmentation compared to WT. The selected colonies were grown in microtiter in liquid F/2 medium and the chlorophylls and carotenoids content were estimated on whole cells by measuring absorption at 680 and 500 nm, respectively. Colonies with an increased 500/680 nm ratio of at least 10% compared to the average of other colonies were further analysed by pigment extraction and quantification. A Leica DM2500 fluorescence microscope (63 × zoom) was used to determine the cell size. In particular, the cell area was obtained by comparing the cell dimensions with the cell counter chamber grid in which each square is 50  × 50 µm. Seventy five cells were measured for each strain.

### Pigment extraction and analysis

Pigment extraction and analysis were performed as described in [23]: pigments were extracted with 100% DMSO at 60 °C for 24 h in dark conditions and analyzed by HPLC as described in [12]. De-epoxidation index was calculated as described in [48].

### Photosynthetic parameters

NPQ, PSII functional antenna size were measured by using a Dual PAM-100 fluorometer (Walz, Effeltrich, Germany) as described in [23]. Oxygen evolution curves were measured using a Clark-type electrode [23]. Net oxygen production was calculated by subtracting the oxygen consumption in the dark after each measurement at the different actinic lights.

### Biomass and lipid productivity

Biomass and lipid productivity of *S4* compared to WT strain were measured in cells grown in Multi-Cultivator MC1000 System (Photon System Instrument, Czech Republic) at 24 °C under continuous light at 400 µmol photons m^−2^ s^−1^ starting from a cells concentration of 10^6^ cell mL^−1^. At the end of the exponential phase, cells were harvested to evaluated biomass yield expressed as dry weight per volume (g L^−1^) or as dry weight per volume per days (g L^−1^ day^−1^). When indicated in the main text, cells at the stationary phase where pelleted and resuspend in a F/2 medium deplete of nitrogen to induce nitrogen starvation. Nile red staining was performed at the end of the growth curve as previously reported in [23].

To optimize the production of EPA and ketocarotenoids, *S4* cells were growth in the Multi-Cultivator MC1000 System at 24 °C with 3% CO_2_ at different light intensities: 100, 500, 1000 and 2000 µmol photons m^−2^ s^−1^. Moreover, when specified in the main text, 10 g L^−1^ of glucose was added. Fatty acid methyl esters (FAME and in particular EPA) were measured as reported in [23].

### Sequencing analysis

Sequencing of mutant and WT strain was performed as reported in [23]. Single nucleotide polymorphisms (SNPs) reported were identified by crossing the results of three software, GATK [49], Freebayes v1.3 and breseq v0.35.1 [50] and by discarding variants found in both samples. Additional file 2 reports the dataset with all mutations identified, their predicted effect obtained using SNPeff software [51], their Gene Ontology associated terms obtained by Blast2GO [52] and their predicted localization based on HECTAR [53]. Blast2GO GO terms were grouped using the plant slim subset and visualized using REVIGO [54], where dots size correspond to the number of genes related to each GO slim term. Proteins encoded by mutated genes addressed in the main text were manually analysed by BlastP and InterPro to predict the function of the encoded proteins.

## Supplementary Information

**Additional file 1: Table S1.** Dry weight concentration, FAME content per dry weight, astaxanthin fraction on total ketocarotenoid and EPA fraction on total FAME. **Table S2.** Coverage obtained by Illumina sequencing of WT and *S4* mutant strain. **Table S3.** Number of SNPs in *S4* mutant strain. **Table S4.** Lists of mutations that caused stop codon, frameshift variant or alteration of the intron/exon pattern (predicted HIGH effect). **Table S5.** Lists of mutations in genes encoding for protein predicted to be direct to chloroplast. Signals peptide were predicted by HECTAR software. **Figure S1**. PSII functional antenna size: (A) PSII maximum quantum efficiency calculated as (FM-F0)/FM where F0 is the basal chlorophyll fluorescence in the dark and FM is the maximum chlorophyll fluorescence induced by a saturating pulse. (B) Fluorescence induction kinetics in WT and *S4* mutant strain in DCMU-treated cells; (C) PSII functional antenna size reported as 1/τ2/3 (%) calculated from kinetics in (B). Error bars are reported as standard deviation (n = 3). Significant different values are marked with * as determined by *t*-student test (n = 3, p < 0.05). **Figure S2.** Biomass and lipid productivity at the end of the growth curve in medium with N (left column) or without N (right column). Lipid content measured by Nile red staining was reported normalized to dry weight (A, B), cell number (C, D) or on volumetric base (E, F) for cells grown in nitrogen replete (+ N) or in nitrogen deficiency (− N) conditions. Error bars are reported as standard deviation (n = 3). Significant different values are marked with * as determined by *t*-student test (n = 3, p < 0.05). **Figure S3.** Astaxanthin and ketocarotenoids content in *S4* strain in nitrogen replete or nitrogen deficiency growth condition. Astaxanthin and ketocarotenodis are reported as mg per gram of dry weight in nitrogen replete (+ N) or nitrogen deficiency (− N) growth condition. **Figure S4.** FAME composition of *N. gaditana S4* strain compared to WT. FAME composition of WT and *S4* strains are reported as percentage of total FAME for cells grown at 500 μmol m^−2^ s^−1^. Error bars are reported as standard deviation (n = 3). Significant different values are marked with * as determined by *t*-student test (n = 4, p < 0.05). **Figure S5.** Revigo software visualization of all GO slim term found for mutated genes of *S4*. Go slim term are divided in three categories: biological process, molecular function and cellular component. Dots size correlate with the number of genes with a determined GO slim term (max. 13 genes, min. 1 genes).

**Additional file 2: **Dataset with the list of SNPs identified in *S4* mutant strain and features of mutated genes.

## Data Availability

All data generated or analyzed during this study are included in this published article and its additional information files.
