# Supplementary material for: Astaxanthin and eicosapentaenoic acid production by S4, a new mutant strain of Nannochloropsis gaditana
Source: Microb Cell Fact. 2022 Jun 16;21:117. doi: 10.1186/s12934-022-01847-9 (PMC9204945; doi:10.1186/s12934-022-01847-9)

Supplementary Table S1. Dry weight concentration, FAME content per dry weight, astaxanthin fraction on total ketocarotenoid and EPA fraction on total FAME

	g DW L^{-1}	ASTA/KETO (%)	FAME (mg g DW^{-1})	EPA (%FAME)
**100 µmol m^{-2} s^{-1}**	0.91 ± 0.09[a,b,c]	53.59 ± 0.76% [a]	209,10 ± 26,94 [a]	6.29 ± 0.08%[a,b]
**100 µmol m^{-2} s^{-1} + 10 g L^{-1} Glu**	1.01 ± 0.15 [a]	50.85 ± 4.64% [a]	269,69 ± 31,30 [a]	6.86 ± 0.42% [b]
**500 µmol m^{-2} s^{-1}**	0.87 ± 0.02 [a,c]	50.76 ± 3.66 [a]	241,23 ± 65,18 [a]	5.18 ± 0.32 [c]
**500 µmol m^{-2} s^{-1} + 10 g L^{-1} Glu**	1.03 ± 0.14 [a,b]	47.91 ± 6.64 [a]	252,05 ± 86,24 [a]	5.30 ± 0.17% [c]
**1000 µmol m^{-2} s^{-1}**	0.81 ± 0.04 [a,c]	54.01 ± 0.64 [a]	264,09 ± 32,33 [a]	6.06 ± 0.77% [a,b,c]
**1000 µmol m^{-2} s^{-1} + 10 g L^{-1} Glu**	1.11 ± 0.15 [b]	55.17 ± 0.49 [a]	229,17 ± 4,96 [a]	5.48 ± 0.33% [a,c]
**2000 µmol m^{-2} s^{-1}**	0.72 ± 0.03 [c]	51.66 ± 0.85 [a]	212,20 ± 19,72 [a]	5.36 ± 0.25% [c]
**2000 µmol m^{-2} s^{-1} + 10 g L^{-1} Glu**	0.91 ± 0.04 [a,b,c]	54.22 ± 0.60% [a]	240,79 ± 25,40 [a]	5.22 ± 0.36 % [c]

Dry weight concentration, FAME content per dry weight, astaxanthin (ASTA) fraction on total ketocarotenoid (KETO) and EPA fraction on total FAME for cells grown in different light regimes with or without glucose (Glu). Errors are reported as standard deviations are reported (n =4).

Supplementary Table S2. Coverage obtained by Illumina sequencing of WT and *S4* mutant strain.

	WT	*S4*
**mean coverage**	115.02	84.66
**% 1X cov**	98.79	98.81
**% 5X cov**	98.47	98.65
**% 10X cov**	98.01	98.20
**% 20X cov**	96.92	96.32

Supplementary Table S3. Number of SNPs in *S4* mutant strain

	Total SNPs	Predicted effect			
		HIGH	MODERATE	LOW	MODIFIER
**SNP in *S4***	199	6	60	39	94

The predicted effect for the different SNPs was retrieved by using SNPeff software, which classify the mutations as HIGH, MODERATE, LOW, or MODIFIER. HIGH are mutations predicted to have high (disruptive) impact in the protein inducing for example the gain of a stop codon, frameshift variant or alteration of splicing site; MODERATE are non-disruptive variants that might change protein effectiveness; LOW are mutations harmless or unlikely to change protein behaviour; MODIFIER are SNPs with predicted non-coding variants, thus localized downstream or upstream genes, in 3' or 5' UTR region or in intergenic region.

Supplementary Table S4. Lists of mutations that caused stop codon, frameshift variant or alteration of the intron/exon pattern (predicted HIGH effect).

#CHROM	POS	REF	ALT	Annotation	Gene_ID	Sequence Description	predicted targeting category
NG_chr12	315668	G	A	splice_acceptor_variant &intron_variant	Naga_100016g35	---NA---	signal peptide
NG_chr10	599703	C	T	stop_gained	Naga_100021g66	e3 ubiquitin-protein ligase listerin	other localisation
NG_chr02	709602	GC	G	frameshift_variant	Naga_100022g21	hypothetical protein PHYSODRAFT_255846 [Phytophthora sojae]	other localisation
NG_chr10	1025719	C	T	splice_donor_variant &intron_variant	Naga_100037g9	ccr4-associated factor	other localisation
NG_chr14	367540	G	A	stop_gained	Naga_100047g12	mj1332 ygr210cp-like gtp binding protein gtpase obg family plus rna binding domain tgs	other localisation
NG_chr13	547291	G	A	stop_gained	Naga_100062g8	mediator of rna polymerase ii transcription	other localisation

Supplementary Table S5. Lists of mutations in genes encoding for protein predicted to be direct to chloroplast. Signals peptide were predicted by HECTAR software.

#CHROM	POS	REF	ALT	Annotation	Annotation_Impact	Gene_ID	Sequence Description	signal peptide cleavage site
NG_chr11	493292	C	T	missense_variant	MODERATE	Naga_100005g23	glutamate synthase	42
NG_chr06	779646	C	T	missense_variant	MODERATE	Naga_100015g35	predicted protein [Thalassiosira pseudonana CCMP1335]	27
NG_chr02	1014961	C	T	missense_variant	MODERATE	Naga_100050g23	carotenoid cleavage dioxygenase 4	24
NG_chr12	97184	G	A	missense_variant	MODERATE	Naga_100146g1	---NA---	51

Supplementary Figure S1. PSII functional antenna size: (A) PSII maximum quantum efficiency calculated as $(F_M-F_0)/F_M$ where F_0 is the basal chlorophyll fluorescence in the dark and F_M is the maximum chlorophyll fluorescence induced by a saturating pulse. (B) Fluorescence induction kinetics in WT and *S4* mutant strain in DCMU-treated cells; (C) PSII functional antenna size reported as $1/\tau 2/3$ (%) calculated from kinetics in (B). Error bars are reported as standard deviation (n=3). Significant different values are marked with * as determined by *t*-student test (n=3, $p < 0.05$).

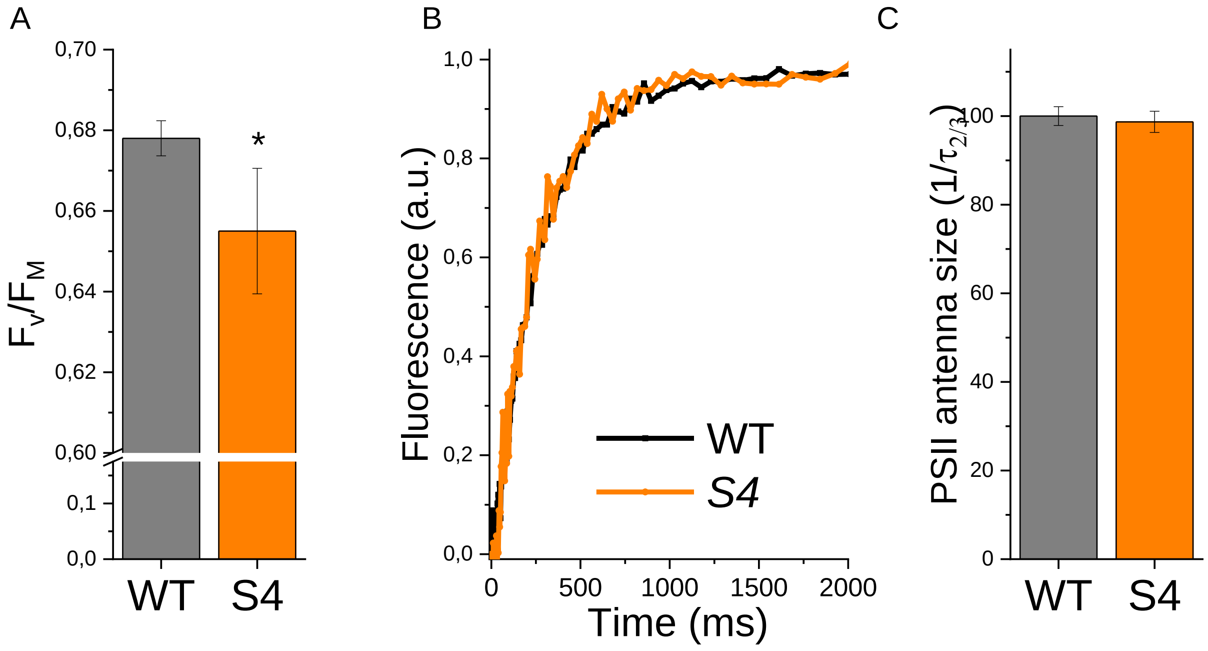

Supplementary Figure S2. Biomass and lipid productivity at the end of the growth curve in medium with N (left column) or without N (right column). Lipid content measured by Nile red staining was reported normalized to dry weight (A, B), cell number (C, D) or on volumetric base (E, F) for cells grown in nitrogen replete (+N) or in nitrogen deficiency (-N) conditions. Error bars are reported as standard deviation (n=3). Significant different values are marked with * as determined by *t*-student test (n=3, p < 0.05).

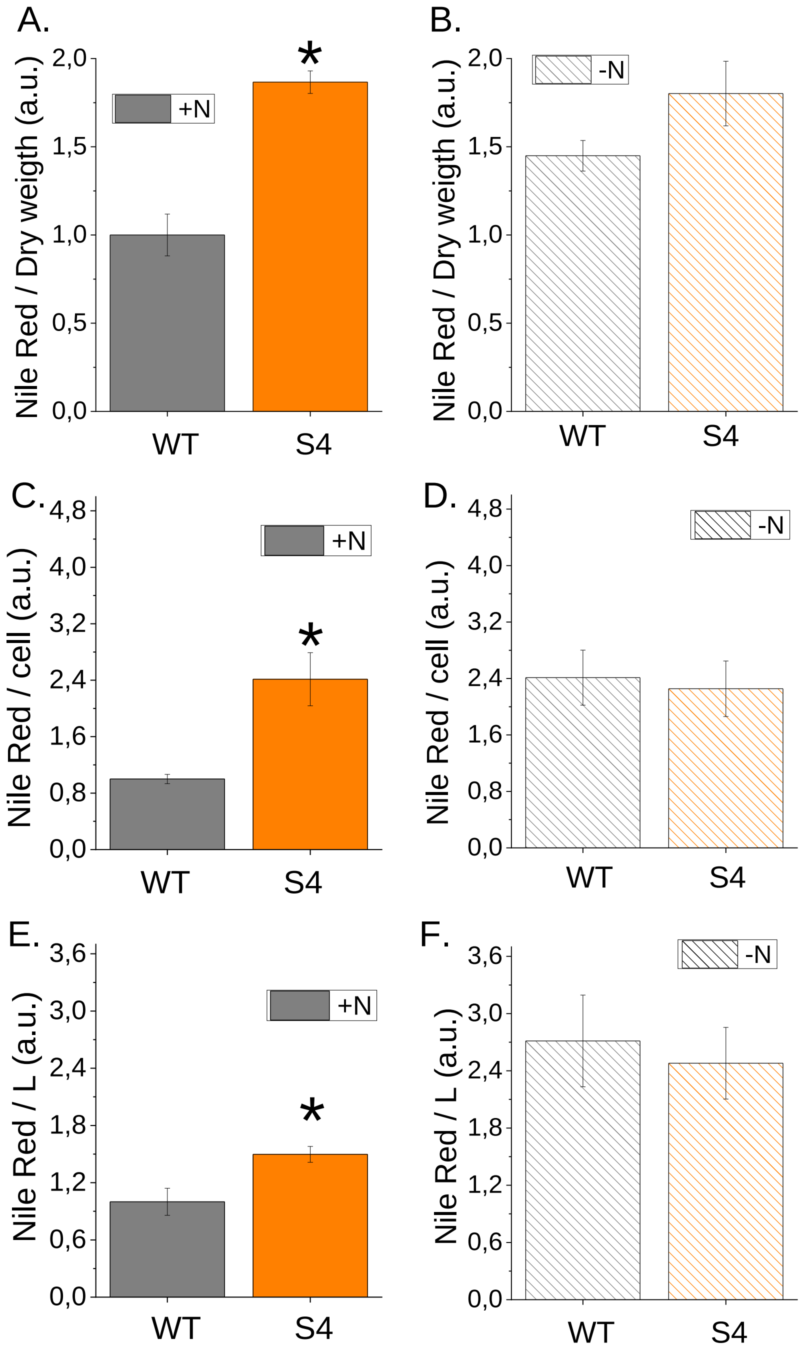

Supplementary Figure S3. Astaxanthin and ketocarotenoids content in *S4* strain in nitrogen replete or nitrogen deficiency growth condition. Astaxanthin and ketocarotenodis are reported as mg per gram of dry weight in nitrogen replete (+N) or nitrogen deficiency (-N) growth condition.

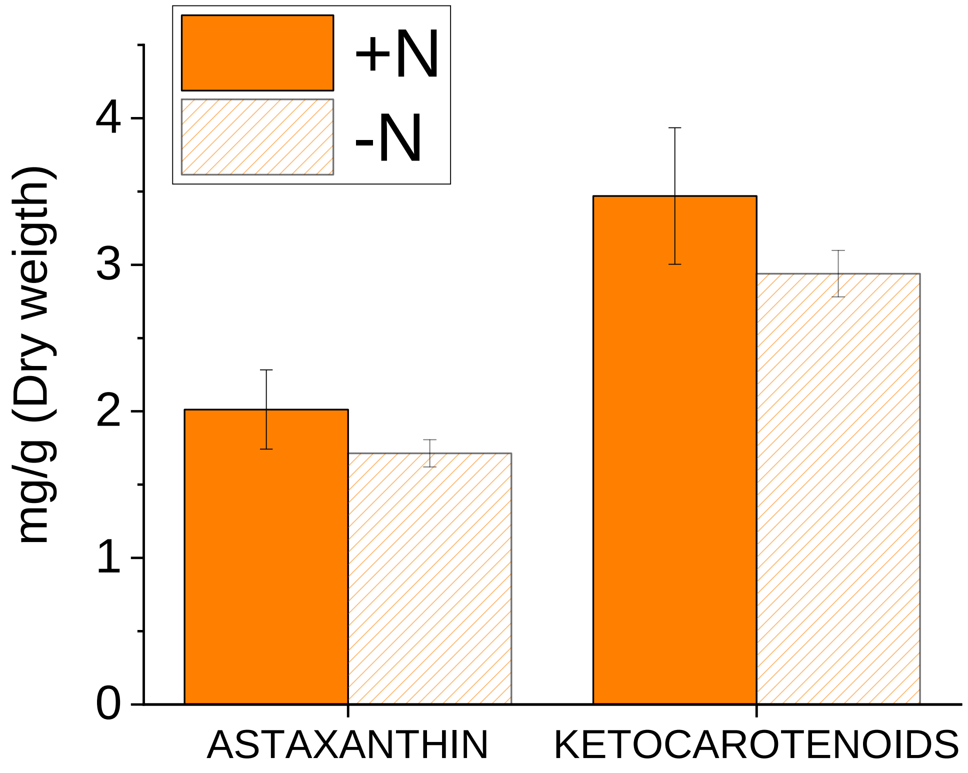

Supplementary Figure S4. FAME composition of N. gaditana *S4* strain compared to WT. FAME composition of WT and *S4* strains are reported as percentage of total FAME for cells grown at 500 µmol m^{-2} s^{-1}. Error bars are reported as standard deviation (n=3). Significant different values are marked with * as determined by *t*-student test (n=4, $p < 0.05$).

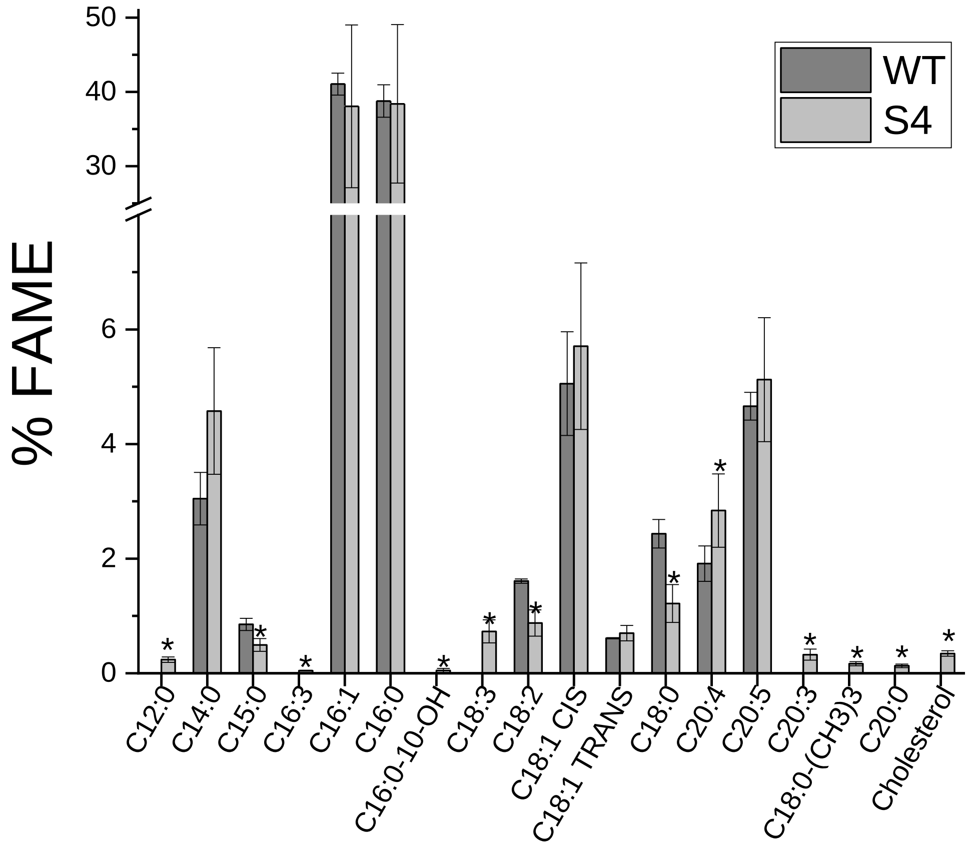

Supplementary Figure S5. Revigo software visualization of all GO slim term found for mutated genes of *S4*. Go slim term are divided in three categories: biological process, molecular function and cellular component. Dots size correlate with the number of genes with a determined GO slim term (max 13 genes, min 1 genes).

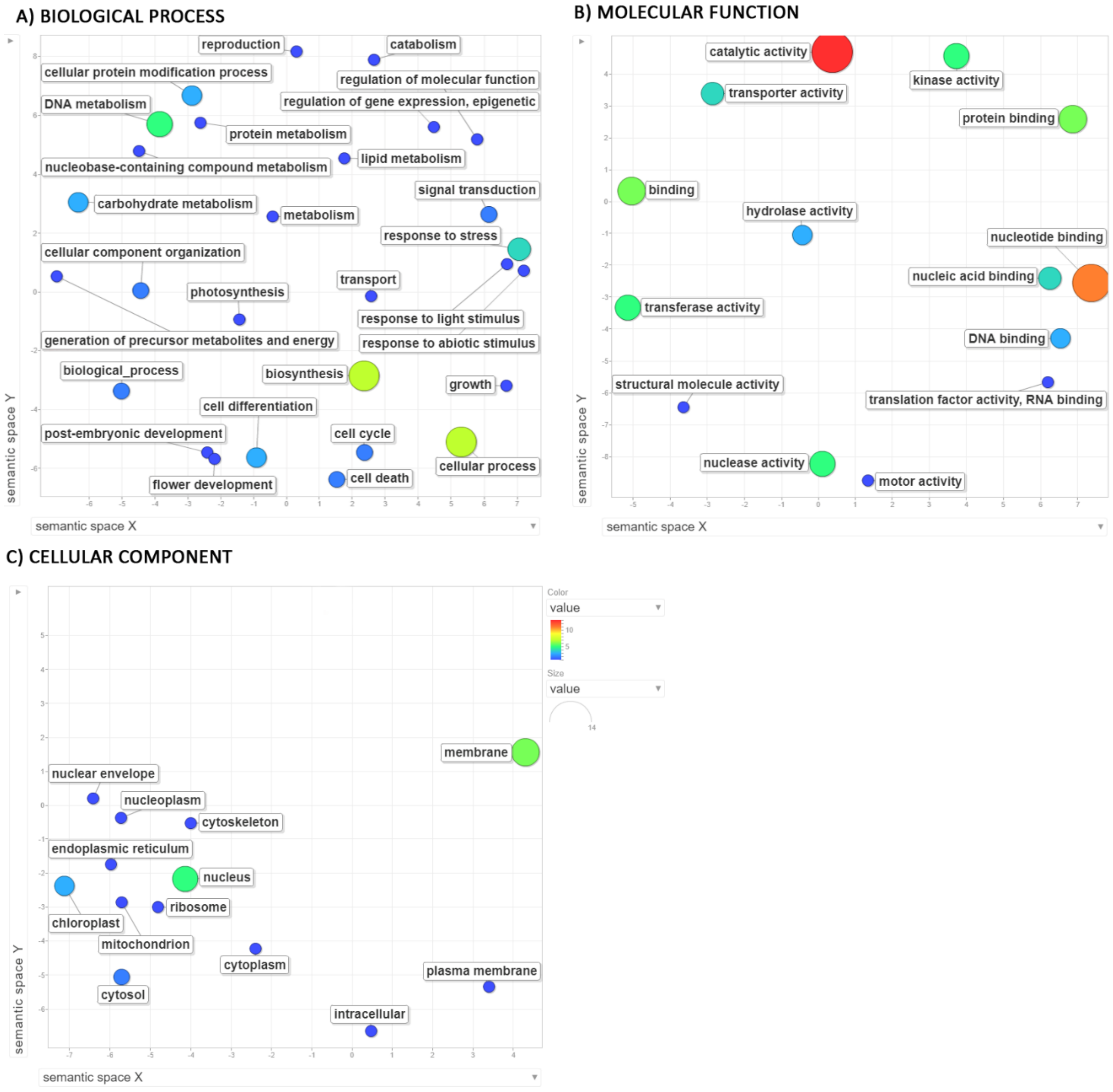

Supplement: Supplementary file 1 — Additional file 1: Table S1. Dry weight concentration, FAME content per dry weight, astaxanthin fraction on total ketocarotenoid and EPA fraction on total FAME. Table S2. Coverage obtained by Illumina sequencing of WT and S4 mutant strain. Table S3. Number of SNPs in S4 mutant strain. Table S4. Lists of mutations that caused stop codon, frameshift variant or alteration of the intron/exon pattern (predicted HIGH effect). Table S5. Lists of mutations in genes encoding for protein predicted to be direct to chloroplast. Signals peptide were predicted by HECTAR software. Figure S1. PSII functional antenna size: (A) PSII maximum quantum efficiency calculated as (FM-F0)/FM where F0 is the basal chlorophyll fluorescence in the dark and FM is the maximum chlorophyll fluorescence induced by a saturating pulse. (B) Fluorescence induction kinetics in WT and S4 mutant strain in DCMU-treated cells; (C) PSII functional antenna size reported as 1/τ2/3 (%) calculated from kinetics in (B). Error bars are reported as standard deviation (n = 3). Significant different values are marked with * as determined by t-student test (n = 3, p < 0.05). Figure S2. Biomass and lipid productivity at the end of the growth curve in medium with N (left column) or without N (right column). Lipid content measured by Nile red staining was reported normalized to dry weight (A, B), cell number (C, D) or on volumetric base (E, F) for cells grown in nitrogen replete (+ N) or in nitrogen deficiency (− N) conditions. Error bars are reported as standard deviation (n = 3). Significant different values are marked with * as determined by t-student test (n = 3, p < 0.05). Figure S3. Astaxanthin and ketocarotenoids content in S4 strain in nitrogen replete or nitrogen deficiency growth condition. Astaxanthin and ketocarotenodis are reported as mg per gram of dry weight in nitrogen replete (+ N) or nitrogen deficiency (− N) growth condition. Figure S4. FAME composition of N. gaditana S4 strain compared to WT. FAME comp [file 12934_2022_1847_MOESM1_ESM.pdf]
